# Our 7-year experience supporting the Ross autograft with the novel technique of Personalized External Aortic Root Support

**DOI:** 10.1016/j.xjtc.2024.02.004

**Published:** 2024-02-14

**Authors:** Ana Redondo, Conal Austin

**Affiliations:** Congenital Cardiac Surgery Department, Evelina London Children's Hospital, Guy's and St Thomas' NHS Foundation Trust, London, United Kingdom

**Keywords:** Ross, aortic root, aortic valve, Personalized External Aortic Root Support

## Abstract

**Objective:**

The Ross operation is a widely accepted option for aortic valve replacement in children, and evidence shows its excellent results in terms of hemodynamics and durability. However, indications are still limited due to the fact that it is a technically demanding procedure, only performed by specialized surgeons. On top of that, and despite numerous techniques being applied, autograft dilatation remains a key disadvantage, which can lead to graft failure. In recent years, the ExoVasc Personalized External Aortic Root Support (PEARS) has proven to be a safe and effective option to prevent aortic root dilatation in various aortopathies and is a technique that lends itself to support the pulmonary autograft in the Ross operation.

**Methods:**

During the past 7 years, we have used the ExoVasc PEARS graft, manufactured from the patients' pulmonary artery measurements from computed tomography scan data, to support the pulmonary autograft in the Ross operation. This graft (manufactured by Exstent Ltd, UK) is implanted at the same time as the autograft. We have reviewed all the patients who underwent this surgery, including demographic data, aorta measurements, operative data, and follow-up assessment consisting of periodic echocardiograms and magnetic resonance imaging scans.

**Results:**

Fifty patients were included in the study. Mean age at the time of the operation was 29.84 years, the youngest patient was 9 years-old. Nineteen patients (38%) had previous sternotomies; 11 of them having had a previous aortic valve replacement. Seventy-two percent of patients had initially a bicuspid aortic valve. Mean diameter of the ascending aorta was 3.83 cm. Forty-four percent of patients required a concomitant reduction aortoplasty due to mismatch sizes between the ascending aorta and the autograft. Mean bypass and crossclamp times were 200.66 and 151.14 minutes, respectively. Median length of stay was 6 days. Mean follow-up was 16.88 months. Two patients required subsequent aortic valve replacement (1 had rheumatic valve disease and the other had iatrogenic damage in his autograft valve leaflet). Ascending aorta dimensions remain stable when compared with immediate postoperative studies. There were no deaths.

**Conclusions:**

The ExoVasc PEARS graft has proven to be an excellent support in the Ross operation to prevent the autograft failure related to autograft dilatation that can offer several advantages compared with other existing techniques. With this type of support, we believe the Ross indications can be expanded to multiple clinical scenarios, given the good long-term results this operation offers in terms of durability, life expectancy, and hemodynamics.

## Graphical abstract

**Figure:**
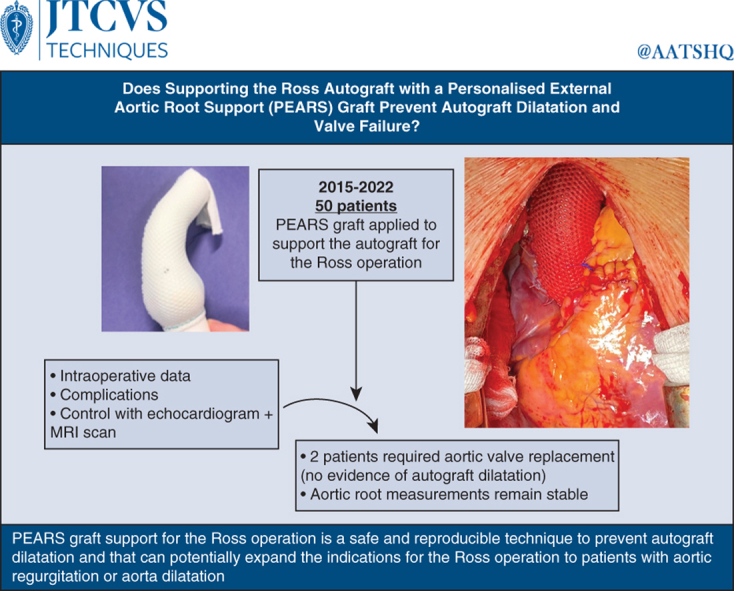

**Figure:**
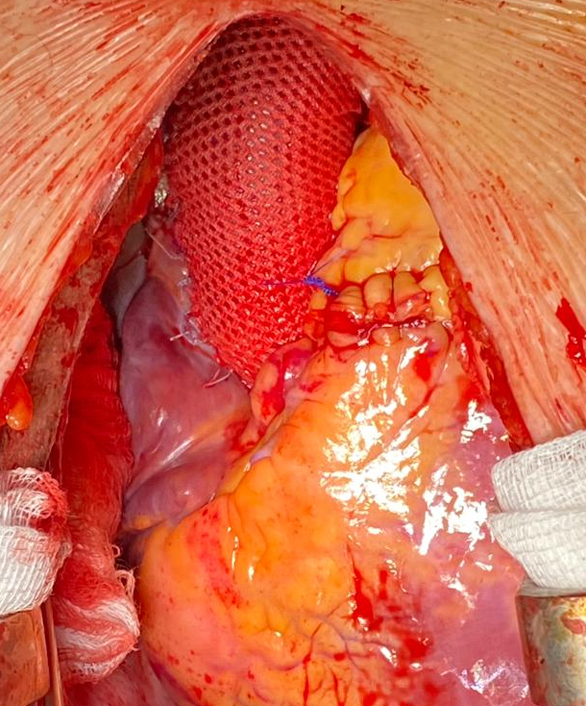
Surgical picture after having performed a Ross-PEARS operation.

**Central Message**

Supporting the Ross autograft with Personalized External Aortic Root Support can prevent dilatation which can lead to potential valve failure. This is a safe and reproducible technique.

**Perspective**

Ross procedure indications can be expanded if a correct support for the autograft is provided. We believe Personalized External Aortic Root Support is an excellent option that, in our experience, has prevented aortic root dilatation. This would allow the Ross operation to be applied in multiple and complex clinical scenarios such as repeat aortic valve surgery or aortic root dilatation.

The Ross procedure was developed in the 1960s^1^ and since then has been the procedure of choice for aortic valve replacement in children and even young adults. For older patients, despite other options, such as mechanical or biological valves, it has progressively gained popularity and one of the main reasons for this is the evidence that it offers major advantages when compared to prosthetic aortic valve replacement. These advantages include better hemodynamics, no need for anticoagulation therapy, reduced risk of endocarditis, and an equivalent survival when compared with the general population.^2^

However, the Ross operation is a more technically demanding procedure, generally associated with longer operative times, and usually performed in small numbers or by highly experienced surgeons and centers.^3^ For all those reasons, there is still a large number of surgeons who are reluctant to perform this operation as a first option.

Furthermore, there are mid- to long-term complications that are typically associated with the Ross procedure, and that make it a less appealing option in many scenarios. First of all, the failure of the homograft or the right ventricle to pulmonary artery conduit, which may require reinterventions for replacement or transcatheter procedures. And secondly and the most feared complication: The autograft failure. The most common approach for the Ross operation is free root implantation, that, without any kind of autograft support, may end up in autograft dilatation and a concerning valve failure due to leaflet coaptation problems in 10% to 20% of patients at 10 to 15 years.^4^

To overcome this, several techniques exist to support the autograft: the so-called inclusion technique sitting the pulmonary artery within the patient's native aorta, polyethylene terephthalate cylinder autograft wrapping, external aortic annulus support suturing, or even a reabsorbable sinotubular junction support suture.^5^

In our institution we have been successfully using the Personalized External Aortic Root Support (PEARS) technique since 2013 (although the first PEARS operation for a patient with Marfan syndrome was performed in 2004) for prevention of aortic dilatation in a variety of genetic aortopathies, including dilated aortas related to congenital heart disease. This technique consists of a personalized polyethylene teraphtalate macroporous mesh created from the exact measurements of the patient's aortic computed tomography (CT) scan, which provides a soft and pliable support for the aortic root. It has a strong proximal hem which is externally anchored to the aortic annulus that prevents its dilatation.^6^^,^^7^ Given the good results offered by the PEARS technique,^8^ our institution was the first one to apply this graft as a support for the autograft in the free-root Ross operation. In this article, we aim to present the results of this combined technique from its inception in 2015.

## Materials and Methods

### Patients

We have included all the patients in our institution who underwent a Ross procedure supported by a PEARS graft (Ross-PEARS). The first patient had this procedure done in 2015. Since then, 50 patients have been included in our database.

The same surgeon performed the operation for all patients, and there was always a surgical indication for aortic valve replacement, which was discussed and agreed in a multidisciplinary meeting. Only Ross operations supported with a PEARS prosthesis were included in this study.

Follow-up consisted of a surgical review in the outpatient clinic 6 weeks after discharge to assess the clinical situation, and then an adult congenital cardiology review, either in our center or in the patients' referral unit, 3 months after discharge (unless any different indication), with a routine echocardiogram to assess the pulmonary and neoaortic valve function and the changes in ascending aorta dimensions. Magnetic resonance imaging (MRI) was done in most cases 1 year after the Ross-PEARS procedure, unless clinical contraindication (permanent pacemaker). After that, an echocardiogram was performed yearly, and an MRI if considered necessary, every 2 years. Only data from the latest echocardiogram and MRI were recorded in the database.

Data were collected retrospectively and recorded in a database. Reports and results were obtained from the hospital's electronic patient record system. Data were requested for those patients who were referred from other units and followed-up externally.

Patients signed a specific and detailed informed consent form with the details of this operation, and they were aware of the novelty of this technique and the possibility of inclusion of their data in research databases. This procedure was also reviewed and discussed in the patient safety group and the TRaQ (Trust Risk and Quality) committee meetings, and is under continual and independent review at our institution. No formal institutional review board review was required because it is a retrospective data collection and analysis.

### Data Collection

Preoperative data were obtained from referral forms, preoperative reports, and multidisciplinary meeting reporting forms. Imaging and CT scan, MRI, and echocardiogram reports were also reviewed. For the aorta and pulmonary artery measurements, the greatest diameter in centimeters for the aortic annulus, sinuses of Valsalva, sinotubular junction, ascending aorta at the level of the right pulmonary artery, pulmonary annulus, and main pulmonary artery were collected. All the CT scans were done under the same Ross-PEARS protocol by the same specialized radiology team. Once the images of the CT scan were sent for manufacturing, the production of the graft usually took around 3 weeks.

The echocardiograms were performed and reported by a specialized adult congenital heart disease team. Following the same criteria, the valvular stenosis and regurgitation was classified in less than mild (trivial or trace), mild, moderate, or severe. The ventricular function was also classified in normal, mildly, moderately, and severely impaired. Images were double reviewed and went through the same classification process as those cases transferred from other units.

### Operative Technique

All cases were performed via midline sternotomy. Bypass was established with central cannulation (proximal aortic arch, superior vena cava, and inferior vena cava). In some cases, the autograft was harvested under induced ventricular fibrillation before the instillation of cardioplegic solution.

Once the heart was arrested with cold blood cardioplegia, the aorta was opened, and the native aortic valve or the previous prosthesis was excised. In cases of small aortic valve annulus with associated narrowed left ventricular outflow tract, an enlargement of the annulus was performed in the following way: after excision of the aortic root, an incision was then made into the ventricular septum down the commissure between the right and the left sinuses of Valsalva, cutting toward the pulmonary outflow; this was continued into the base of the heart until the annulus was sufficiently enlarged, but not creating any ventricular septal defect. Then coronary buttons were harvested. The proximal hem of the PEARS graft was marked with 3 equidistant Prolene stitches, corresponding to the three valve commissures. The axial seam was opened.

The pulmonary autograft was sutured to the aortic annulus with the invagination technique, using a continuous running suture line of 4–0 Prolene, which incorporates both the autograft and the hem of the PEARS graft simultaneously. This is believed to completely reinforce the aortic annulus. The coronary buttons were reimplanted in the autograft at an appropriate level, through 2 apertures cut in the posterior and anterior leftward sinuses of the PEARS graft.

In cases with high discrepancy between the diameters of the pulmonary autograft and the ascending aorta (due to aortic dilatation), a reduction aortoplasty was performed. This comprised making an excision of the excess tissue in the right lateral aortic wall, which was calculated by measuring the ascending aorta and the autograft diameters with Hegar dilators. A pulmonary valve homograft obtained via the National Heart Valve Bank was then implanted in the pulmonary position, starting with the distal anastomotic suture at the level of the bifurcation of the pulmonary trunk. Following this, the autograft was anastomosed to the ascending aorta. The proximal pulmonary homograft anastomosis can be performed in most cases with the heart beating.

Once the patient was successfully off bypass and hemostasis was achieved, the PEARS graft was closed over the aorta by re-suturing the longitudinal axial seam along the noncoronary aspect of the aorta, with single interrupted Ti-Cron (polyethylene terephthalate) stitches. The length of the PEARS graft can be trimmed distally as desired, but it is advisable to go over the whole autograft and ascending aorta, especially after a reduction aortoplasty has been done.

### Statistical Analysis

Descriptive data are used with no statistical analysis. Frequency is described in absolute number and in percentages. For any data analysis, Stata version 14 was used (StataCorp).

## Results

### Patient Characteristics

Fifty patients were included in the study. Main principal characteristics are reflected in Table 1. Median age at surgery was 26 years (range, 9-54 years). Fourteen percent of the patients (n = 7) were aged 18 years or younger. Nineteen patients (38%) were redo sternotomies, 11 of them having undergone previous aortic valve replacements with current prosthetic failure (22%). One patient had a previous Ozaki repair with indication for aortic valve replacement due to repair failure. Seventy-two percent of patients had a background diagnosis of bicuspid aortic valve. The principal current lesion was stenosis in 30% of patients, regurgitation in 22%, and mixed aortic valve lesion in 24%. Mean maximum aortic valve annulus size was 2.53 cm (mean *z* score, +1.97; minimum, −1.67 and maximum, +6.42), mean maximum diameter of sinus of Valsalva, sinotubular junction, and ascending aorta were 3.40 (mean *z* score, +1.67), 3.11 (mean *z* score, +2.3), and 3.83 cm (mean *z* score, +3.18), respectively. Mean maximum size of pulmonary valve and diameter of pulmonary artery were 2.64 and 2.91 cm, respectively. Left ventricular function was normal in most cases (91.30%), whereas 1 patient had, preoperatively, moderately impaired left ventricular function.

**Table 1:** Preoperative patient characteristics

Patient characteristics	n	Result
Age at surgery (y)		26 (20-36)
Male sex	26	52
Reoperation∗	19	38
Initial aortic lesion		
Stenosis	29	58
Regurgitation	8	16
Mixed	9	18
Endocarditis	2	4
Bicuspid aortic valve	36	72
Current aortic lesion		
Stenosis	15	30
Regurgitation	11	22
Mixed	12	24
Prosthetic failure	11	22
Repair failure	1	2
Grade of aortic stenosis		
N/A	11	22
None	3	6
Mild	8	16
Moderate	7	14
Severe	21	42
Grade of aortic regurgitation		
N/A	11	22
None	1	2
Mild	15	30
Moderate	6	12
Severe	17	34
Left ventricular function		
Normal	46	92
Mildly impaired	3	6
Moderately impaired	1	2

Results values are presented as median (interquartile range) or %. *N/A*, Not available.

∗ Number of previous sternotomies ranging 1 to 4.

### Intraoperative Data

Intraoperative data are presented in Table 2. The first 2 cases were performed with a different technique from the one that is currently used as the standard, using the inclusion technique to provide further external support to the PEARS. From then on, all patients underwent a free-root Ross supported only by the PEARS graft. Eight patients had in addition an aortic annulus enlargement. Forty-four percent of the patients had a reduction aortoplasty to facilitate the match between the ascending aorta and the pulmonary autograft. Five patients had concomitant procedures such as mitral valve repair or septal myectomy. Mean crossclamp time was 151.14 minutes, and mean cardiopulmonary bypass time was 200.66 minutes.

**Table 2:** Intraoperative data

Intraoperative data	Result
Type of operation	
Free-root Ross-PEARS	80 (n = 40)
Inclusion Ross-PEARS	4 (n = 2)
Free-root Ross-Konno-PEARS	16 (n = 8)
Reduction aortoplasty	44 (n = 22)
Concomitant procedures (n)	
Mitral valve repair/LAVV repair	2
Septal myectomy	2
Plication of ventricular septal aneurysm	1
Cardiopulmonary bypass time	200.66 ± 8.59
Crossclamp time	151.14 ± 4.96

Values are presented as %, total values, or mean ± SD. *PEARS*, Personalized External Aortic Root Support; *LAVV*, left atrioventricular valve.

### Early Postoperative Outcomes

There were no deaths during the early postoperative period. Three patients among the first 6 in our series, had chest re-exploration within the first 24 hours, 2 of them for suspected coronary injury based on ischemic electrocardiogram changes: 1 had partial kinking of the proximal left anterior descending artery due to proximity of the suture line of the right ventricle to pulmonary artery conduit anastomosis; the other patient had no compromise of the coronary arteries and the electrocardiogram eventually normalized. The third patient was reopened for postoperative bleeding.

Two patients required permanent pacemaker due to complete atrioventricular block, both having had a previous aortic valve replacement. One patient required a postoperative percutaneous coronary intervention with a left main stem stent. This patient had undergone an aortic root replacement for endocarditis 15 years before, presenting with complete heart block, and the coronary ostia were heavily calcified. Median postoperative stay length was 6 days, ranging from 4 to 60 days (the longest was a patient with severe scoliosis and restrictive respiratory function considered moderate to high risk for cardiac surgery, and who required long-term invasive ventilation and tracheostomy).

Postoperative results are reflected in Table 3. All patients had a routine transthoracic echocardiogram before discharge, for assessing the autograft valve and the ventricular function. This showed a mild, or lesser, degree of neoaortic regurgitation in all cases. Regarding the left ventricular function, 2 patients showed mildly impaired function (preoperatively normal, both recovered during follow-up) and 2 showed moderately impaired function (both cases exhibited mild compromised function preoperatively).

**Table 3:** Postoperative data

Postoperative data	Result
Echocardiogram predischarge	
Left ventricular function	
Normal	89.58 (n = 43)
Mildly impaired	4.17 (n = 2)
Moderately impaired	6.25 (n = 3)
Degree of aortic regurgitation	
None	12.50 (n = 6)
Trivial/trace	54.17 (n = 26)
Mild	33.33 (n = 16)
Postoperative stay (d)	6 (4-60)
Follow-up echocardiogram	
Left ventricular function (%)	
Normal	85.71
Mildly impaired	8.57
Moderately impaired	5.72
Degree of aortic regurgitation	
None	21.21
Trivial/trace	45.45
Mild	24.24
Moderate	9.09
Degree of pulmonary regurgitation	
None	45.71
Trivial/trace	28.57
Mild	22.86
Moderate	2.86

Values are presented as % or median (interquartile range).

### Late Postoperative Outcomes

Mean follow-up was 16.88 months. The longest follow-up was nearly 7 years (83.9 months). No patient died during this period of time. In 2 patients follow-up was interrupted because they required aortic valve replacement due to autograft failure: the first (who was the first patient in our cohort), had a rheumatic valve disease, which ultimately influenced the autograft, causing severe regurgitation. The second case was a complex patient with 3 previous complex cardiac operations, and the autograft damage was considered iatrogenic, occurring 3 months later due to left coronary leaflet damage from internal knot of Prolene in the subcoronary area.

**Figure 1:**
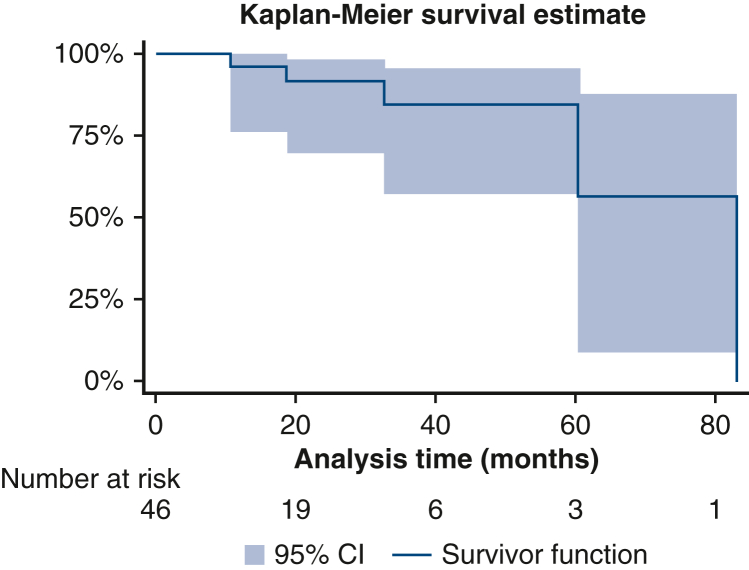
Kaplan-Meier survival curve for events (reoperation for aortic valve regurgitation or discovery of aortic valve regurgitation greater than mild). *CI*, Confidence interval.

**Figure 2:**
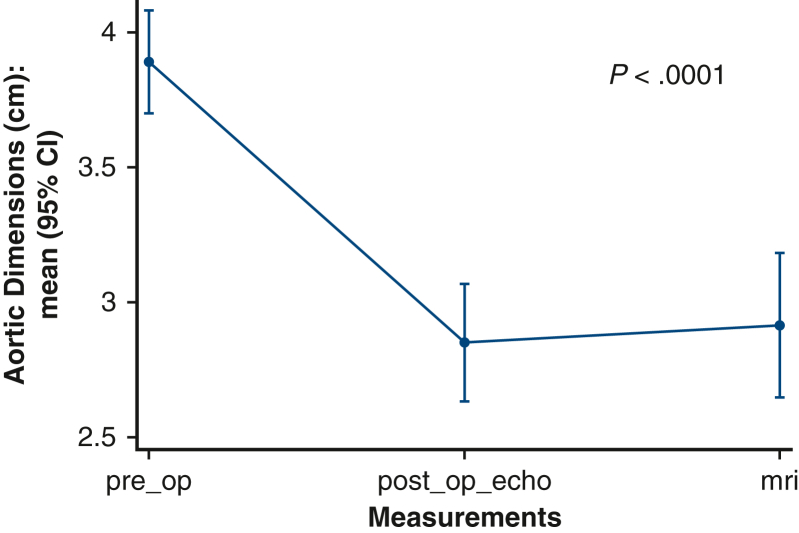
Box plot graphic reflecting the maximum measurements obtained for the aortic root and ascending aorta in the preoperative (*preop*) imaging (echocardiogram [*echo*] or computed tomography scan), in the follow-up magnetic resonance imaging (*MRI*) scan and in the follow-up echo. A general reduction of the diameters can be seen postoperatively compared with the preoperative values. *CI*, Confidence interval; *postop*, postoperative.

All patients had a transthoracic echocardiogram performed within a specialized adult congenital heart disease unit. Apart from the 2 patients who required aortic valve replacement, 3 showed more than mild aortic regurgitation: 2 of them had a later MRI that confirmed the regurgitation was actually not greater than mild, and the third has no current indication for intervention at the moment because she remains asymptomatic. The composite curve for Ross failure (mortality, autograft valve insufficiency, and need for aortic valve replacement) curve is reflected in Figure 1. In all cases, the pulmonary homograft is well functioning and no patient has required reintervention for autograft dilatation as evidence by echocardiogram and MRI scans showed no significant change on aortic root and ascending aorta dimensions (mean maximum aorta dimension during follow-up, 2.84 ± .32 cm); mean *z* score, +0.2 (minimum, −3.6 and maximum, 2.57) (comparative dimensions are in Figure 2).

## Discussion

The Ross operation has some advantages that makes it an excellent option compared to a prosthetic aortic valve replacement, as it is a “living” valve, offering at the same time better hemodynamics and avoiding some other problems, for example anticoagulation-related adverse effects and endocarditis.^9^ For this reason, its indications and uses have been expanded and there is good evidence of its safety in other scenarios outside the classical indications, such as aortic regurgitation and bicuspid aortic valves.^10^^,^^11^

In terms of complications related to this procedure, the pulmonary homograft failure with subsequent need for pulmonary valve replacement is a well-documented adverse event,^12^ although we have not found this type of complication in our series. Other studies also highlighted the low incidence of homograft dysfunction, especially when using decellularized homografts.^13^ Furthermore, in those cases, the advanced percutaneous techniques would allow replacement of the valve with no need for a repeat sternotomy.^14^

Autograft failure, on the other hand, is a more feared complication that can lead to valve failure. This seems to be directly related to autograft dilatation when this is implanted as an unsupported free-root because it is exposed to systemic pressures compared with pulmonary pressures. Autograft failure can be as frequent as 40% to 60%.^15^ The free-root Ross is the most widespread Ross technique due to its surgical simplicity, compared with other techniques such as the subcoronary Ross, which is considered more challenging because it risks losing the structure of the valve and offers less support to the annulus.^16^

The latest trends for improving the Ross operation have been, unsurprisingly, focused on supporting the autograft to prevent its dilatation. Several techniques have been described in the literature: from the inclusion technique^17^ (which we used in the past) to annular external reinforcement suture or polyethylene terephthalate cylinder graft autograft wrapping.^18^ However, our experience has shown that the PEARS graft offers some important advantages compared with these other options. First of all, it adapts perfectly to the patients' anatomy, thanks to the personalized manufacturing process and the soft and pliable material used. In fact, the importance of preserving the aortic root hemodynamics has been highlighted on numerous occasions.^19^ Second, we have experienced that it is technically less complex compared with, for example, the inclusion technique, especially for the coronary transfer. Also, the fact that it is a macroporous mesh and that it is implanted as an open graft, allows a better inspection of the autograft to achieve hemostasis and the graft is only closed over the aorta once any bleeding has been resolved. And lastly, the PEARS graft can provide support for all the ascending aorta up to the midaortic arch in cases of aortic dilatation related to, for example, bicuspid aortic valve disease.

The development of the PEARS-supported Ross has been a project in evolution. As such, the development and size of the PEARS prosthesis has evolved in the following way: Our past Ross technique was the inclusion, which is for highly selected patients without significant aortic insufficiency nor asymmetry or dilatation of the aortic root. The first 2 patients in this cohort had an inclusion Ross, with the PEARS manufactured on the size of the aorta. After that, we changed to a free-root Ross with a PEARS prosthesis made to match the size of the pulmonary artery. Patients 3 through 8 had a 100%-sized PEARS graft manufactured by this technique; however, at implantation it was found that the graft was tight. The reason for this was we believe that the CT scan images the internal lumen of the vessel, and does not take into account the thickness of the pulmonary artery wall and its associated fatty adventitia. Subsequently, we ordered 120% prosthesis (a 20% uniform increase in size) for the following 5 patients; this was eventually found to be too big. Cases 14 and 15 had a 105% prosthesis manufactured, and finally after case 16 we settled on a 115% prosthesis, which is now the mainstay of our PEARS support for the Ross operation.

The results are very promising. In the only 2 cases where the autograft failed, there was no evidence of autograft dilatation (*z* score of the autograft root in the last measurements were −0.46 and −0.91) leading to the valve failure, rather than a leaflet issue causing the problem. These cases are among the first 8 cases in our cohort (the ones receiving the 100%-sized graft).

The first patient was, in fact, our first failure because he had severe aortic insufficiency on the basis of previous rheumatic disease. The pulmonary autograft failed within 6 months and the leaflets of the autograft retracted, leading us to confirm that patients with rheumatic heart disease are not good candidates for the Ross operation because the pulmonary valve can be similarly affected, which has also been reported by other groups.^20^ The second autograft failure was also an early patient in the series and he had a left coronary leaflet tear that occurred after 3 months when an internal subcoronary knotted suture split the leaflet. Both patients had mechanical aortic replacements in a similar way to a failed biological root replacement.

The technique of a PEARS-supported Ross surgery we believe will enable consideration to be given to those cases traditionally believed to be poor Ross candidates; that is, aortic root and ascending aorta dilatation with severe aortic valve insufficiency. Furthermore, in cases where there is a disparity between annulus sizes of the aortic compared with the pulmonary, the PEARS proximal hem that is incorporated into the proximal autograft suture line would stabilize and reduce the annulus to that of the slightly enlarged autograft. Ross-PEARS also is very applicable to resolving failed biological and mechanical aortic valve prosthesis as seen in more than 20% of our cohort.

The operative times and postoperative hospital stays compare favourably with those for simple free-root Ross procedure, which suggests that the PEARS application does not add any more difficulty to the operation. Furthermore, in both cases that required reintervention, it was found that the PEARS graft did not pose an extra challenge in terms of adhesions. Nevertheless, we have to recognize that this is a technically demanding procedure that has a learning curve and it has to be done by experienced surgeons who are familiar with the Ross operation, appropriately proctored by experienced Ross-PEARS surgeons.

Our experience is still limited to 50 cases, but we also have increasing numbers of patients being referred (either from our own unit, from other units all over the world, or even self-referred) because more clinicians become more familiar with the technique.

There are some limitations to this study. First of all, we have not been able to compare this cohort to our previous Ross patients treated before 2015. Most of these earlier patients had the inclusion technique and, therefore, the inclusion criteria for having the operation was very strictly limited. With Ross-PEARS we have expanded the indications, including patients with dilated aorta, severe valve regurgitation, or previous aortic valve replacement, so we consider the 2 groups are not comparable.

Also, the fact that a significant number of patients have been referred from other parts of the country, or even other parts of the world, can make follow-up more challenging. Although we try to unify the surveillance protocol, this cannot always be properly followed, especially considering the pandemic global situation we all endured in the past 3 years.

Furthermore, we need to point out that the initial aortic measurements were mostly obtained from the CT scan, whereas the follow-up imaging is based on echocardiogram and MRI measurements, which could impact in some way the dimensions obtained. We still advocate for follow-up MRI imaging to avoid excess doses of radiation in this relatively young cohort of patients. We recommend a more unified study strategy, with multiple collaborations with units around the world that are interested in adopting this technique, and that can provide the data to assess results in an effective and reliable way.

## Conclusions

The supported Ross procedure is gaining popularity and we have worked on a method that we believe simplifies the support and makes the operation more reproducible. Our experience with PEARS for dilatational aortopathies led us to work with Exstent Ltd to produce an anatomical support for the autograft based on a CT pulmonary angiogram.

The PEARS support is incorporated into the proximal suture line and it covers the autograft and the ascending aorta to the level of the proximal aortic arch. However, it is still a technically complex technique performed by experienced surgeons, mostly congenital disease specialized.

We believe that this application of exact anatomical support for the autograft in the Ross will expand the role of Ross operations into more complex cases, for example aortic valve regurgitation with root and ascending aorta dilatation, and unfavorable commissure geometry that besets inclusion techniques. This technique does not add any extra complexity to the operation, and in our initial experience of 50 patients, it has offered good results, maintaining stable neoaortic root and ascending aortic dimensions.

### Webcast

You can watch a Webcast of this AATS meeting presentation by going to: https://www.aats.org/resources/our-7-year-experience-supporting-the-ross-autograft-with-the-novel-technique-of-the-personalised-external-aortic-root-support.

**Figure:**
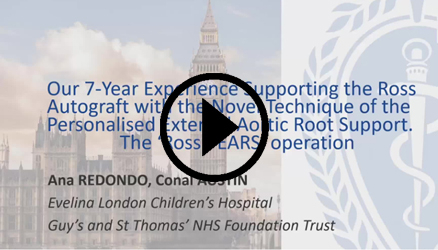

## Conflict of Interest Statement

The authors reported no conflicts of interest.

The *Journal* policy requires editors and reviewers to disclose conflicts of interest and to decline handling manuscripts for which they may have a conflict of interest. The editors and reviewers of this article have no conflicts of interest.
